# Vascular endothelial growth factor A amplification in colorectal cancer is associated with reduced M1 and M2 macrophages and diminished PD-1-expressing lymphocytes

**DOI:** 10.1371/journal.pone.0175563

**Published:** 2017-04-12

**Authors:** Katharina Burmeister, Luca Quagliata, Mariacarla Andreozzi, Serenella Eppenberger-Castori, Matthias S. Matter, Valeria Perrina, Rainer Grobholz, Wolfram Jochum, Daniel Horber, Peter Moosmann, Frank Lehmann, Dieter Köberle, Charlotte K. Y. Ng, Salvatore Piscuoglio, Luigi Tornillo, Luigi M. Terracciano

**Affiliations:** 1 Institute of Pathology, University Hospital Basel, Schönbeinstrasse 40, Basel, Switzerland; 2 Institute of Pathology, Cantonal Hospital Aarau, Tellstrasse 25, Aarau, Switzerland; 3 Institute of Pathology, Cantonal Hospital St. Gallen, Rorschacher Strasse 95, St. Gallen, Switzerland; 4 Medical Oncology and Hematology, Cantonal Hospital St. Gallen, Rorschacher Strasse 95, St. Gallen, Switzerland; 5 Medical Oncology, Cantonal Hospital Aarau, Tellstrasse 25, Aarau, Switzerland; 6 Division of Gastroenterology, University Hospital of Basel, Schönbeinstrasse 40, Basel, Switzerland; 7 Medical Oncology, St. Claraspital, Kleinriehenstrasse 30, Basel, Switzerland; 8 GILab AG, Lettenweg 118, Allschwil, Switzerland; University of Nebraska Medical Center, UNITED STATES

## Abstract

VEGFA is an angiogenic factor secreted by tumors, in particular those with *VEGFA* amplification, as well as by macrophages and lymphocytes in the tumor microenvironment. Here we sought to define the presence of M1/M2 macrophages, PD-1-positive lymphocytes and PD-L1 tumoral and stromal expression in colorectal cancers harboring *VEGFA* amplification or chromosome 6 polysomy. 38 CRCs of which 13 harbored *VEGFA* amplification, 6 with Chr6 polysomy and 19 with neutral *VEGFA* copy number were assessed by immunohistochemistry for CD68 (marker for M1/M2 macrophages), CD163 (M2 macrophages), programmed death 1(PD-1)- tumor infiltrating and stromal lymphocytes as well as tumoral and stromal PD-1 ligand (PD-L1) expression. CRCs with *VEGFA* amplification or Chr6 polysomy were associated with decreased M1/M2 macrophages, reduced PD-1-expressing lymphocyte infiltration, as well as reduced stromal expression of PD-L1 at the tumor front. Compared to intermediate-grade CRCs, high-grade CRCs were associated with increased M1/M2 macrophages and increased tumoral expression of PD-L1. Our results suggest that *VEGFA* amplification or Chr6 polysomy is associated with an altered tumor immune microenvironment.

## Introduction

The complex interactions between tumor cells and non-tumoral cells within the tumor microenvironment contribute to the hallmarks of cancer cells [1]. The tumor microenvironment is composed of many different cell types including endothelial cells, pericytes, fibroblasts and immune cells [1]. The recent promising results of PD-1/PD-L1 blockade as an immunotherapy check-point in different cancer entities [2–5] have underscored the essential role of the immune system in the control of tumor growth.

Tumor-associated macrophages (TAMs) are found within tumors as well as in the surrounding non-malignant tissues [6] and can be either pro- or anti-tumorigenic in response to environmental changes [7–9]. Macrophages are broadly classified into two major groups, M1 and M2. M1 macrophages are involved in inflammatory response, pathogen clearance and antitumor immunity through the expression of pro-inflammatory cytokines such as IL-1β, IL-6, IL-12, IL-23, TNFα and nitric oxide synthase 2 (iNOS) [6,10–13]. By contrast, M2 macrophages are known to promote tissue remodeling and repair, angiogenesis and tumor progression [14,15]. M2 macrophages release anti-inflammatory cytokines such as IL-10 and transforming growth factor β (TGFβ) and are characterized by an upregulation of mannose receptors (e.g. CD206) and arginase-1, and a downregulation of iNOS production [10,16,17]. The prognostic implication of the extent of macrophage infiltration is uncertain in colorectal carcinomas (CRCs) with reports variably showing associations with favorable prognosis [18] and with adverse prognosis [19] but is generally associated with poor prognosis in other cancer types [20,21]. The contradictory results may be associated with the type and localization of macrophages in the tumor and/or with macrophage infiltration at the tumor front [18].

Activated T-cells and other immune cells typically show upregulation of programmed cell death-1 (PD-1), which plays an immune-suppressive role when bound to its ligand PD-L1 [2]. PD-L1 is expressed by T and B cells, dendritic cells, macrophages, endothelial, muscle and pancreatic cells [22] and its upregulation in cancer cells has been implicated in shutting down immune response in cancer cells [22]. The interaction between PD-1 and PD-L1 results in the downregulation of lymphocyte proliferation and cytokine production [23]. Tumor infiltrating PD-1-positive T-cells and tumoral expression of PD-L1 have been associated with poor prognosis in several tumors, including esophageal, pancreatic, gastric, hepatocellular, urothelial and renal cell carcinomas, follicular lymphoma, melanoma as well as soft tissue sarcomas [23–33]. However, the role of PD-1/PD-L1 in CRC is controversial [22,34]. The contradictory results may be caused by technical limitations, as well as by the heterogeneity and variability of these markers which are strongly affected by temporal and spatial factors [34], leading to different interpretation when detected in different sections of the same tumor.

Recently, we showed that a subgroup (~7%) of highly aggressive CRCs harbor copy number amplification of vascular endothelial growth factor A (*VEGFA*), a member of the vascular endothelial growth factor (VEGF) family, which also includes VEGFB, VEGFC, VEGFD and placental growth factor (PlGF) [35]. VEGFs have been shown to play multi-faceted roles in stimulating neo-angiogenesis and tumor growth [36] and among the VEGFs, VEGFA, in particular, has been shown to mediate angiogenesis, a critical step in both tumor growth and metastasis formation [2,37]. In fact, VEGFA is a key regulator of proliferation, survival, migration and permeability of blood endothelial cells in both physiological and pathological angiogenesis [2,37]. Consistent with these results, copy number amplification and overexpression of *VEGFA* have been associated with poor prognosis in various cancer types [38–40]. In addition to its well-documented angiogenic roles, VEGFA has also been shown to have immunosuppressive properties, including the inhibition of dendritic cell maturation and T-cell production [41,42]. In fact, a recent study demonstrated that VEGFA produced in the tumor microenvironment directly increases PD-1 expression on intratumoral CD8+ T-cells and combined anti-PD-1 and anti-VEGFA blockade showed a synergistic effect in tumors with high levels of VEGFA [43]. It is also important to note that in addition to tumor cells, macrophages and, to a lesser extent, tumor infiltrating lymphocytes (TIL), represent major sources of VEGFA, and macrophage-produced VEGFA has been shown to promote tumor angiogenesis and invasion [44,45]. Thus the interactions between tumors with *VEGFA* amplification, macrophages and PD-1-expressing lymphocytes are likely to be intricate.

To address the question whether there is any association of *VEGFA* copy number status and alterations of the immune microenvironment in CRCs, we performed an immunohistochemical study to define the presence of M1/M2 macrophage (using CD68 and CD163 as markers for M1/M2 and M2 macrophages, respectively), the presence of PD-1-positive tumor infiltrating and stromal lymphocytes and the distribution of PD-L1 expression in the tumor and the stroma. We found that *VEGFA* gene copy number amplification/polysomy was associated with reduced macrophages, PD-1-positive tumor infiltrating lymphocytes and PD-L1 stromal expression.

## Materials and methods

### Ethics

Samples were anonymized prior to analysis and the study has been approved by the Institutional Review Board of the Institute of Pathology, University Hospital Basel (USB), Switzerland, and the Ethics Committee of Nordwest/Central Switzerland (EKNZ). Participants in the USB underwent a written, informed consent process at enrolment.

### Tissue samples

The biobank at the Institute of Pathology, University Hospital Basel, Switzerland, was searched for CRCs diagnosed between 2007 and 2013. In total, formalin-fixed paraffin-embedded (FFPE) samples of 150 CRCs and 45 adjacent non-malignant tissue samples were retrieved. Additionally, whole sections of 8 CRCs previously found to harbor *VEGFA* copy number amplification (n = 2) or chromosome 6 polysomy (n = 6) [35] were retrieved from the Institutes of Pathology of the Cantonal Hospitals of Aarau and St. Gallen, Switzerland.

### Tissue microarray (TMA) construction

All FFPE samples had sufficient material for TMA construction. Hematoxylin and eosin-stained sections were obtained from each FFPE block to define representative tumor tissue regions. TMAs were constructed by punching the regions of interest using core cylinders of 1 mm diameter using TMA-GM^®^ (Sysmex AG, Switzerland). Four-μm-thick slides of the resulting TMAs were cut using Microtome (Thermo Fisher Scientific Inc., USA).

### Fluorescence In Situ Hybridization (FISH)

FISH for *VEGFA* gene copy number status was performed using validated protocols established at the Institute of Pathology at the University Hospital Basel as described previously [35,46]. *VEGFA*-amplified cases were defined as a *VEGFA*/Chr6 ratio of <2.0 and an average *VEGFA* copy number of ≥6.0 signals per cell or a *VEGFA*/Chr6 ratio ≥2.0 with an average *VEGFA* copy number of ≥4.0 signals per cell. Samples with a *VEGFA*/Chr6 ratio of <2.0 and an average *VEGFA* copy number <4.0 signals per cell were classified as not amplified. Samples with a *VEGFA*/Chr6 ratio <2.0 and an average *VEGFA* copy number ≥ 4.0 and <6.0 signals per cell were classified as equivocal. For Chr6 polysomy status, low polysomy 6 was defined as an average between 2.26 and 3.75 Chr6 copy number and high polysomy 6 was defined as an average higher than 3.75 Chr6 copy number [46]. For TMA punches that were positive or equivocal for *VEGFA* amplification or Chr6 polysomy, FISH was performed on a whole slide from the corresponding FFPE block to confirm the positive results or to resolve the equivocal cases [35,46].

### Immunohistochemistry

Whole FFPE sections were pre-treated with CC1 (Ventana Medical Systems, Tucson, Arizona, USA) as previously described [47] and incubated with primary antibodies against CD68 (IR613, Dako, Denmark, pre-diluted), CD163 (Cat. No. 760–4437, Ventana Medical Systems Inc., USA, pre-diluted), PD-1 (Cat. No. 760–4895, Ventana Medical Systems Inc., USA) and PD-L1 (Cat. No. 13684, Cell Signaling Technology, USA). Positive and negative controls were included in each experiment. Immunohistochemistry for each marker was evaluated twice by the same observer (KB) using the BX43 light microscope (Olympus, Japan). Discordant cases were reviewed by two pathologists with a special interest in gastrointestinal pathology (LT and LMT) to reach a consensus. Representative micrographs were acquired using the cellSens Dimension software (Olympus, Japan) and the DP73 Camera (Olympus, Japan) installed on the BX43 light microscope.

For CD68 (M1/M2 macrophage marker) and CD163 (M2 macrophage marker), IHC scoring was performed for 4 randomly selected fields at the tumor front for each case. The number of macrophages was counted for each marker on a total field of 2.2 mm^2^ using the ImageJ program (version 1.46r). Semi-quantitative/ categorical comparisons were also performed using the following thresholds. For CD68-positive macrophages, fewer than 100 macrophages was considered low infiltration, between 100 and 130 moderate infiltration and more than 130 high infiltration. For CD163-positive macrophages, fewer than 60 macrophages was defined as low infiltration, between 60 and 90 macrophages as moderate infiltration and more than 90 macrophages as high infiltration.

For PD-1, 4 random spots at the tumor front of each case were selected and photographed with a 40X objective and a 10X ocular with a total magnification of 400X. A total of 152 pictures in 38 tumors were evaluated. PD-1 expression in tumor infiltrating and stromal lymphocytes were evaluated separately. The number of PD-1-positive cells were counted on a total field of 2.2 mm^2^ using the ImageJ program (version 1.46r). For categorical analyses, the infiltration of positive PD-1 cells per 2.2mm^2^ was scored as no infiltration (0 to 8 cells), low infiltration (9 to 39 cells) and high infiltration (more than 39 cells). PD-1 expression in tumor infiltrating and stromal lymphocytes were evaluable in 36/38 samples.

PD-L1 expression was evaluated using the scoring system as described by Kim *et al*. [23] to evaluate the intensity and the area of staining, separately in tumor and stromal areas at the tumor front. Staining intensity was graded semi-quantitatively as: 0 for negative staining, 1 for weakly positive staining, 2 for moderately positive staining and 3 for strongly positive staining. Area of staining was scored as 0 for 0–10% stained cells, 1 for 11–33% stained cells, 2 for 34–66% stained cells and 3 for 67–100% stained cells. A combined PD-L1 score was defined as the sum of the intensity score and the area of staining score, with a minimum score of zero and a maximum combined score of six. For categorical analyses, total scores were divided into three groups: 0 for no expression, 1 to 2 for low expression, and 3 to 6 for high expression. PD-L1 expression in tumoral and stromal areas were evaluable in 38/38 and 37/38 samples, respectively.

### Statistical analysis

Statistical analyses for categorical and non-categorical variables were performed using Chi-Square/ Fisher’s Exact and Mann-Whitney U/ Student’s t tests as described in the manuscript or figure legends. All tests were two-sided. p-values <0.05 were considered statistically significant. All analyses were performed using Graphpad Prism 6.0 (Graphpad Software, Inc., La Jolla, CA) or R x64 Version 3.2.1 (http://www.R-project.org).

## Results

### *VEGFA* copy number assessment and sample selection

**Fig 1:**
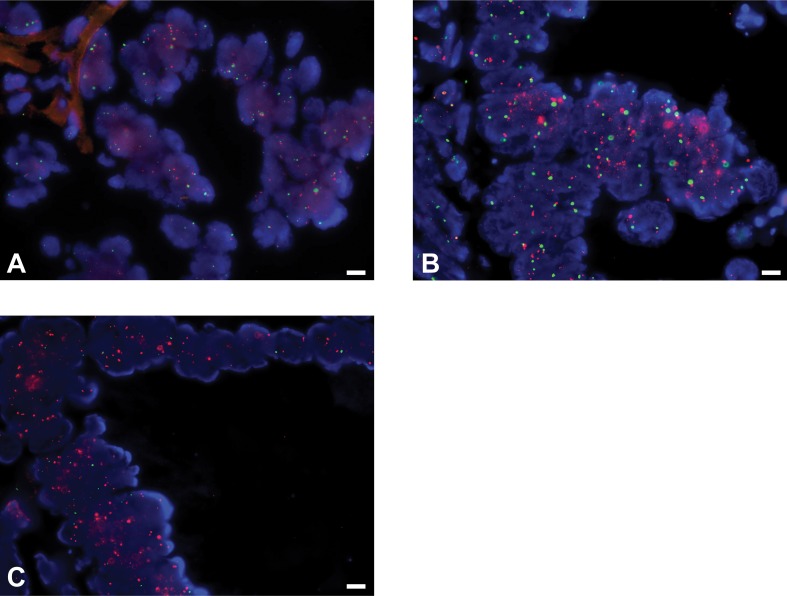
*VEGFA* copy number status in colorectal cancers measured by fluorescent *in situ* hybridization. Representative micrographs of (A) diploid, (B) polysomic and (C) amplified *VEGFA* using fluorescent *in situ* hybridization (FISH). FISH analysis was performed using two-color probes for *VEGFA* (red) and internal control (green). Scale bar 20 μm.

To identify cases of CRC with *VEGFA* gene amplification, we screened a TMA consisting of 150 CRCs by FISH for *VEGFA* gene amplification and/or chromosome 6 (Chr6) polysomy. Of the 124 evaluable samples, we identified 11 samples (9%) with *VEGFA* amplification and none that displayed Chr6 polysomy. The remaining 113 samples (91%) were *VEGFA* copy number neutral. In addition, we retrieved the whole sections from eight cases previously found to harbor *VEGFA* amplification (n = 2) or Chr6 polysomy (n = 6) [35]. In total, we selected the 19 CRCs with *VEGFA* amplification (n = 13) or Chr6 polysomy (n = 6) and the same number of *VEGFA* copy number neutral CRCs (n = 19) as control (Fig 1 and S1 Table). Most the included CRCs were of intermediate tumor grade (i.e. grade 2, n = 29) and the remaining were of high grade (i.e. grade 3, n = 9, S1 Table). The *VEGFA* status stratified on the histologic grade revealed that in the intermediate tumor grade CRCs VEGFA was amplified, polysomic or diploid in 38% (11/29), 14% (4/29) and 48% (14/29) respectively. In the CRCs of high tumor grade VEGFA was amplified, polysomic or diploid in 22% (2/9), 22% (2/9) and 56% (5/29) respectively. Further analysis revealed that there were no differences in sex, T/N/M stage, tumor grade, lymphatic and venous invasion between the *VEGFA* amplified/polysomic and the control group (p>0.05, Chi-square tests).

#### *VEGFA* amplification/polysomy is associated with reduced M1 and M2 macrophages

**Fig 2:**
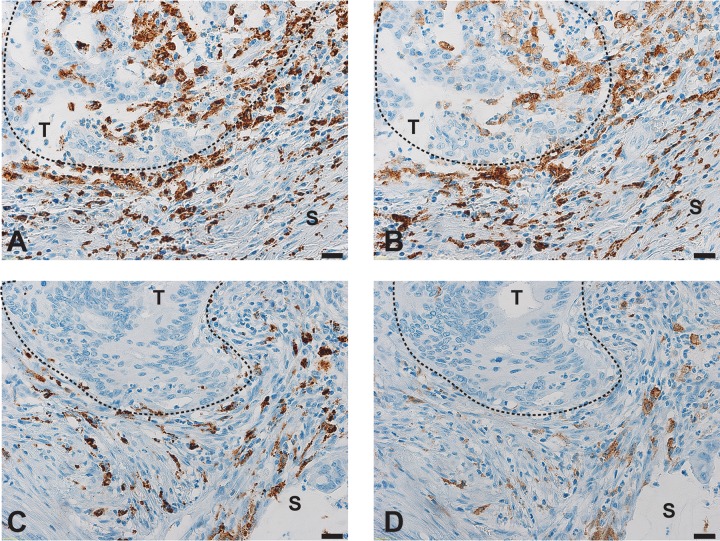
Distribution of macrophages in colorectal cancers, using CD68 and CD163 markers. Representative micrographs of (A, C) CD68+ cells and (B, D) CD163+ cells (A, B) at the invasive tumor front (T) and (C, D) in the surrounding tumor tissue (S). Magnification 40X. Scale bar 20 μm.

To determine the distribution of macrophages in CRCs, we performed immunohistochemical analysis using CD68 as a marker for both M1 and M2 macrophages and CD163 as a marker for M2 macrophages [6]. In the 38 CRCs included in our cohort, both CD68-positive cells and CD163-positive cells were almost exclusively located in the tissue surrounding the tumors, especially along invasive tumor front (Fig 2). Semi-quantitative evaluation of CD68 and CD163 expression at the tumor front revealed that 16 (42%), 13 (34%) and 9 (24%) CRCs had low, moderate and high infiltration of CD68-positive cells, respectively, and 33 (87%), 3 (8%) and 2 (5%) CRCs had low, moderate and high infiltration of CD-163-positive cells, respectively (S2 Table).

**Fig 3:**
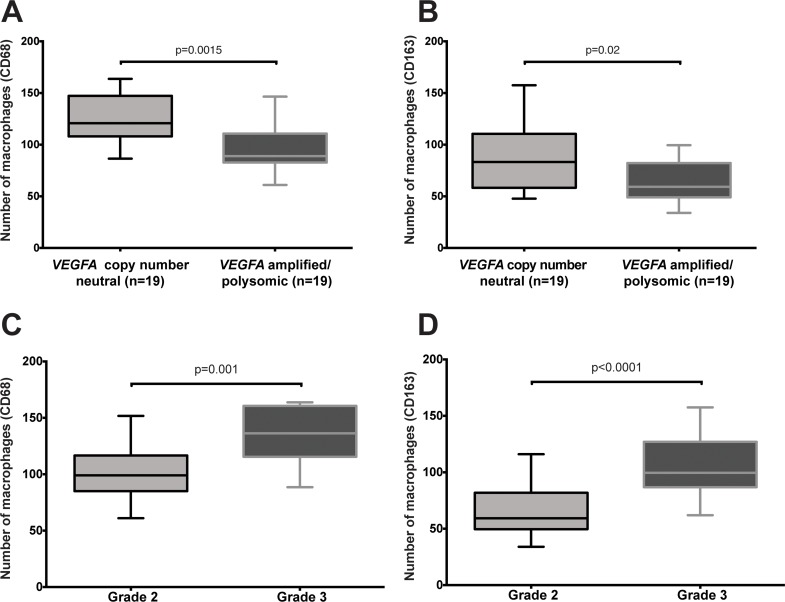
Number of macrophages in colorectal cancers, using CD68 and CD163 markers. Boxplots depict the number of (A, C) CD68+ and (B, D) CD163+ cells (A, B) in CRCs with and without *VEGFA* amplification/polysomy and (C, D) in CRCs of intermediate and high grade.

Statistical analysis between the number of CD68- and CD163-positive cells with and without *VEGFA* copy number amplification/ polysomy revealed reduced CD68-positive cells in CRCs with *VEGFA* gene amplification or Chr6 polysomy compared to those that were *VEGFA* copy number neutral (p = 0.0015, Mann–Whitney *U* test; Fig 3A). When we categorized the number of CD68-positive cells into low, moderate and high infiltration, a predominantly low infiltration (p = 0.0139, Chi-square test, S2 Table) was found in CRCs harboring *VEGFA* gene amplification or Chr6 polysomy. Similarly, we observed fewer CD163-positive cells in CRCs with *VEGFA* gene amplification/ Chr6 polysomy than *VEGFA* copy number neutral CRCs (p = 0.02, Mann–Whitney *U* test; Fig 3B). However, there was no statistical difference we categorized the number of CD163-positive cells into low, moderate and high infiltration (p = 0.218, Chi-square test, S2 Table). We further observed that both CD68 and CD163 infiltration were increased in high-grade tumors (grade 3) compared to intermediate-grade tumors (grade 2, p = 0.001 and p<0.0001, respectively, Mann–Whitney *U* tests; Fig 3C and 3D and p = 0.002 and p<0.0001, respectively, Chi-square test, S2 Table). The increased CD-163 positive cells are M2 macrophages. The fraction of M1 macrophages was on the contrary reduced in high-grade cases.

Taken together, these results suggest that CRCs with *VEGFA* gene amplification or Chr6 polysomy are associated with reduced M1/M2 (CD68-positive) and M2 (CD163-positive) macrophage infiltration, whilst high-grade CRCs are associated with increased M2 and reduced M1 macrophage infiltration.

#### *VEGFA* amplification/polysomy is associated with reduced PD-1-positive tumor lymphocytes and PD-L1 stromal expression

Next we investigated the association of *VEGFA* gene copy number status with the presence of PD-1-positive tumor infiltrating and stromal lymphocytes and PD-L1 tumoral and stromal expression at the tumor front. In this cohort, we observed no PD-1-expressing tumor infiltrating lymphocytes and PD-L1 tumoral expression in 50% and 66% of the CRCs analyzed. By contrast, PD-1 positive lymphocytes were present and PD-L1 was expressed in the stroma in 89% and 92% of cases respectively (S2 Table).

**Fig 4:**
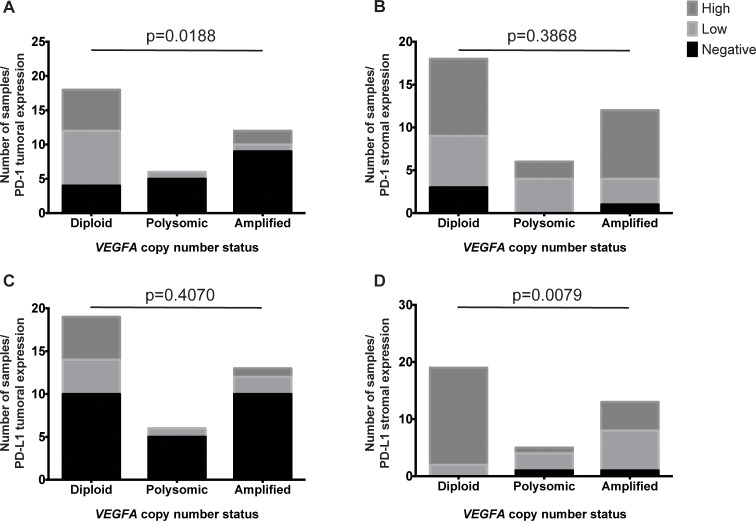
PD-1-positive tumor infiltrating and stromal lymphocytes and PD-L1 tumoral and stromal expression in colorectal cancers. Barplots depict the number of samples with high, low and negative expression of (A, B) PD-1 and (C, D) PD-L1 in (A, C) tumoral and (B, D) stromal areas of CRCs.

Similar to the reduction in M1 and M2 macrophages, CRCs with *VEGFA* amplification or Chr6 polysomy were preferentially associated with the absence of or the reduction in PD-1-positive tumor infiltrating lymphocytes than in *VEGFA* copy number neutral CRCs (p = 0.0188, Chi-square test; Fig 4A), but were not different from *VEGFA* copy number neutral CRCs in terms of the number of PD-1-positive stromal lymphocytes (p = 0.3868, Chi-square test; Fig 4B). We further observed no difference in PD-L1-tumoral expression between CRCs with *VEGFA* amplification or Chr6 polysomy and *VEGFA* copy number neutral CRCs (p = 0.407, Chi-square test; Fig 4C). However, we observed that CRCs with *VEGFA* amplification or Chr6 polysomy had lower PD-L1 expression in the stroma than in *VEGFA* copy number neutral CRCs (p = 0.0079, Chi-square test; Fig 4D). Neither PD-1-positive tumor infiltrating nor stromal lymphocytes was associated with tumor grade (p = 0.4355 and p = 0.839, respectively, Chi-square tests; S2 Table). However, increased tumoral but not stromal PD-L1 expression was associated with high-grade (grade 3) compared to intermediate-grade tumors (grade 2, p = 0.0173 and p = 0.4743, respectively, Chi-square test; S2 Table).

Taken together, these results suggest that CRCs with *VEGFA* gene amplification or Chr6 polysomy are associated with reduced PD-1-positive tumor infiltrating lymphocytes and PD-L1 stromal expression.

## Discussion

To understand the immune microenvironment of CRCs with *VEGFA* gene copy number amplification or Chr6 polysomy, we performed a hypothesis-generating immunohistochemical analysis and found an association between *VEGFA* gene copy number amplification or Chr6 polysomy and reduced number of M1 and M2 macrophages, reduced PD-1-expressing lymphocyte infiltration, as well as reduced stromal expression of PD-L1 at the tumor front. We further observed a higher number of M2 macrophages and increased PD-L1 tumoral expression in high-grade tumor compared with intermediate-grade CRCs.

In accordance with Forssell *et al*. [18], we observed that most CD68+ and/or CD163+ macrophages were found in the stroma along the tumor front. This suggests that macrophages are attracted to or recruited to the invasive front. In high-grade tumors these were mostly CD163+, M2 macrophages. These results are in keeping with similar data recently obtained in other neoplastic entities as gastric and lung carcinoma [48,49]. In addition, M2 macrophage polarization has been reported to be significantly associated with advanced histopathologic stage and the presence of metastasis, probably mediated by Caspase recruitment domain-containing protein 9 (CARD9) through activation of the nuclear factor-kappa B signaling pathway [50]. Moreover, we further found that *VEGFA* gene copy number amplification or Chr6 polysomy, a feature that characterizes a subset of aggressive CRCs, was associated with reduced TAMs. These results are in agreement with the previous report linking high CD68+ macrophage infiltration at the tumor front of CRC to improved survival of patients [18]. By contrast, this subset of aggressive CRCs is also associated with lower levels of PD-1-positive tumor infiltrating lymphocytes but high levels of PD-1-positive tumor infiltrating lymphocytes have been robustly associated with poor survival in many cancers [23,26,30–32]. Unfortunately due to the limited number of cases, we were unable to perform survival analysis for this cohort.

VEGFA has previously been associated with immunosuppression in tumors [41,42]. In addition to being produced by a substantial proportion of tumors and being over-expressed when amplified [38–40], VEGFA is also secreted by various cell types, including macrophages and lymphocytes, in the tumor microenvironment. Our observations led to the hypothesis that *VEGFA* amplification may suppress the attraction of macrophages and lymphocytes towards the tumor front. We speculate that pre-angiogenic tumor tissues, which do not harbor the *VEGFA* gene copy number amplification and therefore express low levels of VEGFA, send signals to the bone marrow and/or the blood circulation that lead to the recruitment of the macrophages [51]. Once in the proximity of the tumor, the macrophages release metalloproteinases, such as MMP-9. MMP-9 cleaves the components in the extracellular matrix, releasing VEGFA from its sequestered state and inducing angiogenesis around the tumor, as seen in hyperplastic islet of Langerhans [51]. By contrast, we speculate that the recruitment process of TAMs may not occur in tumors harboring *VEGFA* gene amplification, as the additional VEGFA secreted by surrounding cells may no longer provide a growth advantage. The observation that *VEGFA* amplified/polysomic CRCs had both reduced numbers of CD68-positive and/or CD163+ macrophages and lower PD-L1 stromal expression may further support our hypothesis, since PD-L1 has been reported to be overexpressed in macrophages [51].

Although our cohort in the study was small and the markers used for the identification of macrophages may be imperfect, we found a consistent pattern of macrophages at the tumor front using two markers, in agreement with a previous study [18]. It would be of interest to investigate whether the adverse prognosis associated with the extent of macrophage infiltration in CRC and other cancer types [19–21] overlaps with the aggressive nature of CRCs associated with *VEGFA* amplification.

## Conclusions

In summary, we have identified an association between *VEGFA* gene amplification in CRC and reduced macrophages, PD-1-positive infiltrating lymphocytes and PD-L1 stromal expression at the tumor front. Further studies are needed to clarify the role of VEGFA on the interaction of CRCs and their tumor microenvironment and to provide mechanistic insight into these observations.

## Supporting information

S1 Table Clinico-pathological parameters and VEGFA copy number status of the 38 cases included in the study. (XLS)

S2 Table Immunohistochemical analysis of CD68, CD163, PD-1 and PD-L1 in the cohort. (XLSX)
